# Treatment of Syphilis and the Proper Method of Administering Mercury

**Published:** 1902-01

**Authors:** 


### TREATMENT OF SYPHILIS AND THE PROPER METHOD OF ADMINISTERING MERCURY.

Dr. Winfield Ayres, of Bellevue Hospital, New York, writes on this subject, (*The Lancet*, London, October 19, 1901,) in which after reviewing the various methods heretofore in use for the administration of mercury in the treatment of syphilis, he pronounces unqualifiedly in favor of mercurol, a nucleid of mercury containing 10 per cent, of the metallic element. This form of mercury it will be remembered was introduced by Dr. Carl Schwickerath, of Bonn. His reasons for arriving at this conclusion are, that after conducting a long series of experiments, he finds that mercurol can be given continuously for an unlimited period of time without producing gastric or intestinal disturbance.

Reviewing the methods of administration hitherto in use, Dr. Ayres said in brief: Of the drugs that are frequently used in the treatment of syphilis, I regard blue ointment as of the most value. After it I would place mercurol, bichloride, protoiodide, tannate, blue mass, grey powder, biniodide, fumigations, inhalations, and for extreme cases hypodermic injections of mercury. The unguentum hydrargyri comes to us in the form of metallic mercury minutely subdivided, and it is absorbed directly into the blood as such. A serious objection, however, to administering this form of mercury is that it is extremely nasty, and therefore very few patients can be induced to continue it for any length of time. Of internal preparations, those which are most used are protoiodide and tannate. We apparently get good results in about a third of the cases treated with these preparations; but in the remainder the results are very apt to be disappointing. Everything is frequently found to go well up to a certain point. Then gastro-enteritis sets in, the patient becomes very anemic and generally run down, and a change has to be made to some other medicine. Then we have bichloride. It is very reliable,—much more so than grey powder or protoiodide,—but it causes gastric irritation in a certain number of cases, and it has to be given up and some other preparation substituted.

Dr. Ayres adopts the theory that "in order to exercise its antisyphilitic influence free mercury must circulate in the blood in minutely divided particles." It is known that metallic mercury is not a gastro-intestinal irritant either before its absorption into the blood, or during its excretion; for instance, there is no irritation during its excretion after inunction. It is probable then that mercurol owes its superiority to the fact that its nuclein is not a foreign element, and that therefore no disturbance results from it during the excretion of its constituents.

It is well known that mercury in its pure state has no germicidal properties. We also know that it accumulates—probably by being deposited in the protoplasmic cells in its native state. It has been suggested therefore that the mercury in mercurol is incorporated more readily on account of its association with nuclein which brings it into intimate relation with the cell structures during the process of assimilation, and mercurol probably exerts its influence in some physical way. This theory is at least suggested by clincal experience, for mercurol controls the secondary symptoms of syphilis more readily than any other preparation of mercury and that when given in does containing smaller quantities of the metal. It is moreover important, as already noticed, that when thus administered, the antisyphilitic remedy causes no gastric intestinal irritation.

TREATMENT OF NASAL CATARRH BY THE GENERAL PRACTITIONER. EUGENE C. UNDERWOOD (*St. Louis Medical and Surgical Journal*, July, 1901,) says: I have long entertained the view that the general medical practitioner neglects to treat his patients for catarrh and sends them to a specialist when he could successfully manage these himself. In fact, the treatment of catarrh is very simple and the results which follow correct and systematic treatment are very satisfactory. In practice, two forms of chronic nasal catarrh are met. These are hypertrophic rhinitis and atrophic rhinitis.

The hypertrophic form is more generally seen, and is characterised by a thick mucus discharge from the nose, great liability to colds, obstruction of one or both nostrils, which force the patient to breathe through his mouth, nasal intonation of the voice. There is more or less headache and the sense of smell is lost or impaired. There is dryness of the throat, deafness and other symptoms showing the extension of the disease to neighboring organs. Exostosis of the osseous structures often is seen.

Atrophic rhinitis (ozena) is characterised by a sense of dryness in the nose and throat, a thick, purulent discharge and the expulsion of discolored crusts and an offensive putrid odor. The sense of smell is impaired and the patient is weak and anemic. The mucous membrane is dry and glazed, but in advanced cases ulceration and necrosis are present. The treatment consists of applications directly to the diseased area and the administration of such internal remedies as will correct any coëxisting disease or morbid state. In some cases where there is occlusion by exostosis the resources of surgery must be invoked.

Let me examine more in detail the treatment of the types of nasal catarrh. In simple chronic hypertrophic rhinitis the results of treatment will be most flattering. In a case attended with no constitutional disease nothing is necessary beyond having the patient spray the nasal mucous surface with a solution composed of equal parts of water and hydrozone every three hours.

If the case has persisted some time and the patient has an amount of mucus discharge, I have him take twenty drops of balsam of copaiba four times daily. The hydrozone is not only a disinfectant and germicide, but its curative action on the inflamed mucous membranes is speedy and is not equaled by any other drug I have ever used. When the patient is anemic I have him take iron, and any other drug is used when it is called for by any associated disease or morbid condition, but the hydrozone spray is used in all cases.

In the atrophic variety we shall have to use the same local application. The hydrozone at once overcomes the offensive odor and takes off the purulent crusts. These cases must be treated with cod liver oil, iron and such other remedies as will bring up the general health. Here are a few clinical histories:

Mr. R. H. M., age 60, had been a sufferer for two years. There was no exostosis, but when he had a cold he could breathe only through his mouth. He was in good general health, so I had him buy an atomiser and use a spray composed of equal parts of distilled water and hydrozone. He sprayed the mucous surface of the nose every three hours. On this he made rapid improvement and in three weeks had no further symptoms.

S. M. T., age 18, had chronic hypertrophic nasal catarrh in which the mucus discharge was very abundant, and this was associated with dryness of the throat and constant desire to hawk and spit. She used the hydrozone and water spray, and took fifteen drops of balsam copaiba three times daily. I had the pleasure of seeing this young woman go along to complete recovery in a period of six weeks.

Mrs. R. J. C., age 49. This lady had atrophic rhinitis and as soon as she came near you the putrid odor asserted itself. Her general health was lowered. I had her use the hydrozone and water spray and take cod liver oil internally. She spent last winter in Cuba, and has just gotten home greatly improved in general health and her catarrhal disease is better. She says the spray effectually destroys the disgusting odor and that scarcely any discharge now appears.

